# Women’s Empowerment, Income, and Nutrition in a Food Processing Value Chain Development Project in Touba, Senegal

**DOI:** 10.3390/ijerph19159526

**Published:** 2022-08-03

**Authors:** Cheryl O’Brien, Laura Leavens, Cheikh Ndiaye, Djibril Traoré

**Affiliations:** 1 Department of Political Science, San Diego State University, San Diego, CA 92182, USA; 2 Department of Agricultural Economics, Purdue University, West Lafayette, IN 47907, USA; lleavens@outlook.com; 3 Institut de Technologie Alimentaire (ITA), Dakar 545, Senegal; chndiaye@ita.sn (C.N.); djibril.traore@okstate.edu (D.T.)

**Keywords:** value chain, food processing, women’s empowerment, development, gender, nutrition, dietary diversity, food security, religion, Senegal

## Abstract

To study the impacts of implementing a gender-sensitive value chain development (VCD) initiative in the agri-food sector, we conducted a mixed-methods study of a woman-owned food processing business and its associated value chain in Touba, Senegal. As a result of partnering with a USAID-funded project, the business began producing instant fortified flours, an innovative, higher-value product compared to traditional porridge, using extrusion and fortification techniques. Drawing on Senegalese women’s association networks, the business connected with local women who could work as processors and retailers. Our study’s goal was to explore how the project’s support of this food processing value chain has affected the lives of women processors and retailers, farmers, and medical personnel along the value chain. Particularly relevant to our study is the general lack of opportunities for women to earn their own incomes in the study region, especially outside of the home, and provide for their families. Through surveys, interviews, observations, and novel participatory focus group activities, our study provides qualitative and quantitative evidence of the perceived impacts of value chain development on women’s empowerment, income, and nutrition by key stakeholders in the value chain. We find an often cited barrier to women’s empowerment is the husband’s lack of understanding and limitations placed on women’s mobility, yet we also find perceptions of women’s empowerment in this conservative religious context. Our findings and discussion highlight the need for more research into VCD projects on the complex and, at times, contradictory processes of women’s empowerment. The women in our study expressed a desire for freedom to work outside of the home, and they expressed a need for childcare and contraception. Notably, the women discussed positive community changes, such as infrastructure and the creation of a childcare center, that implicate women’s collective empowerment. We also highlight a promising research opportunity in Senegal to explore the subnational variation in women’s empowerment through VCD.

## 1. Introduction

Agricultural value chain development (VCD) is recognized by many development practitioners as an important market-based strategy for fighting global poverty and food insecurity. The importance of efficient agricultural value chains for poverty reduction is also well-established in the literature [1,2,3,4,5]. Even a decade ago, half of the International Fund for Agricultural Development’s (IFAD) approved projects included an element on VCD [6]. In theory, if the private sector sees that the VCD interventions are profitable, this creates incentives for entrepreneurs to further develop the value chains [7]. However, there is less empirical evidence on the effectiveness of VCD interventions, despite a large amount of attention and the funds they receive [1,2,8,9].

To improve VCD effectiveness, implementing gender-sensitive approaches to VCD is critical. One of the early papers on gender in global value chains explains, “The strength of a value chain approach is its aim to analyze all aspects of design, production and marketing through to consumption, but the totality of that analysis can only be achieved if gender is included as an integral part” [10] (p. 92). Gender-sensitive VCD integrates gender into VCD design, implementation, and assessment. To design more inclusive interventions from a gender-sensitive approach, a value chain analysis would assess: gendered roles and relations in the value chain; gendered access to and control over the assets necessary for value chain participation; gender-differentiated effects of interventions; and gendered power relations’ impacts on actors throughout the value chain [11,12]. Gender-sensitive VCD will likely recommend gender equity measures, given the gendered constraints that can be highlighted through gender-sensitive analysis. Stoian et al. [12] (p. 496) state: “From a development perspective, … careful design and implementation of VCD can provide opportunities to enhance gender equity [13,14]”. For instance, VCD gender-sensitive interventions may “include tailoring of financial products to the needs of women in diverse types of households to facilitate their participation in value chains [15]” [12] (p. 495–496). In another example, “VCD with explicit gender equality goals may focus on strengthening women’s own enterprises, particularly for products traditionally produced by women (e.g., [16])…” [12] (p. 496).

For a VCD that lacks a gender-sensitive approach, Farnworth [11] (p. 3) cautions: “When value chain interventions do not capture gender issues, gender disparities in workloads and incomes may increase, with knock-on effects for human development indicators. Women may also be directly excluded from the benefits of development intervention”. Women, as a social group, face barriers in their value chain participation due to gender norms and unequal power dynamics in their households and communities. Stoian et al. [12] (p. 495) provide a literature review on gender and women in VCD, noting “that value chains are embedded in socio-cultural contexts in which informal gender norms and values, beliefs, and power relations operate across scales–from the household and community levels to the national and global economy. These social norms, relations, and institutions shape women’s and men’s often unequal ability to participate in and benefit from VCD [17]”. For example, women perform the bulk of non-remunerated household labor or reproductive labor, thereby limiting women’s time and energy to participate in value chains; in contrast, men have greater control over assets, decision-making power, and have the capacity to participate in more profitable areas of value chains [14]. Gender-sensitive VCD is important in identifying and addressing gender-based constraints and opportunities within the value chain.

Incorporating gender is especially important when working in agri-food value chains because food production, consumption, and preparation are highly gendered activities [18], thus, included in this gender-sensitive approach, women’s empowerment in the agri-food value chains must be incorporated to improve the outcomes for development projects [19,20]. Studies show that women’s empowerment improves food security, nutrition, and health, while gender inequalities exacerbate food security gaps [21,22,23]. In sum, gender research should inform the VCD projects, including design and policies [24].

To better understand the perceived impacts of implementing a gender-sensitive VCD initiative in the agri-food sector, we conducted a mixed-methods study of a woman-owned food processing business and its associated instant fortified flour value chain in Touba, Senegal. Our goal was to explore the perceptions of how a USAID-funded project’s support of this value chain has affected the lives of women processors and retailers, farmers, and medical personnel along the value chain. This study was not designed to establish causal relationships between the project and its outcomes, but rather to study the perceived impacts of the project through the eyes of the actual beneficiaries by using interviews, participatory activities, observations, and quantitative surveys.

This study contributes to the literature in multiple ways. There is a growing literature on gender in global agri-food value chains, however, much of this literature focuses on production, particularly in the context of export-oriented horticultural crops in sub-Saharan Africa (see [13,25] for review). Far less attention has been paid to the processors and mid-stream actors, those between the farmers and consumers [25,26]. We contribute to this literature by examining the impacts of VCD on women processors and retailers in a non-export-oriented agricultural (instant fortified flour) value chain. In addition, while the majority of our study participants are women processors and retailers, we included other mid-stream actors in our surveys and interviews. As such, our study contributes to the VCD literature by including the perceptions of farmers and medical personnel about their involvement in the project’s instant fortified flours value chain. Specifically, we find that farmers (who are all men) perceive income benefits from their participation in the value chain, while the medical personnel perceive nutritional benefits from the instant fortified flours for their under or malnourished patients.

This study also contributes to the literature on women’s empowerment and nutrition. Women’s empowerment is especially important for children’s nutritional wellbeing, including reducing child malnutrition and children’s overall health [27,28,29,30,31,32,33]. In addition, women’s empowerment is associated with improved household food security and nutrition for all household members [34,35]. Several studies have documented the link between gender inequalities and food insecurities [34,36,37,38,39,40,41,42]. Some studies specifically show that gender inequality in agriculture exacerbates food insecurities [43,44]. We contribute to this literature by providing qualitative and quantitative evidence of the perceived impacts of a VCD project on women’s empowerment as well as household food security and dietary diversity. We document not only the women processors and retailers’ perceptions of women’s empowerment and household nutrition but also the medical personnel’s perception of nutrition based on their participation in the instant fortified flours value chain.

The agricultural development and food security agencies have increased their focus on women’s empowerment to decrease gender inequalities and food insecurities across households. As suggested by gender-sensitive development approaches, women’s empowerment is tied to development donors’ pursuit of gender equality, which demands enhancing women’s empowerment in VCD to help decrease household poverty; this pursuit in VCD led to the creation of measures of women’s empowerment [45,46,47]. One such creation is the Women’s Empowerment in Agriculture Index (WEAI), which is the first comprehensive and standardized measure to capture women’s empowerment and inclusion levels in the agricultural sector [48]. (For details on pro-WEAI, which was still in development at the time of this study, see weai.ifpri.info accessed on 2 May 2022). Inspired by the WEAI’s holistic view of women’s empowerment, we focused our activities and survey questions for women processors and retailers on gathering data that could provide some insights into women’s empowerment from their participation in the VCD project. Due to resource and time constraints as well as recall bias concerns, we were limited with the empowerment questions asked in our survey and through our activities. Nonetheless, we contributed to the gender equality and food security literature by using mixed methods, including novel participatory approaches and quantitative surveys, to assess the perceptions of women’s empowerment in an individual project focused on an instant fortified flour value chain. This contribution includes the perceptions of gender-based constraints in a VCD project of a woman-owned business and its associated women’s network of processors and retailers. “[G]aps persist in coverage of gender-based constraints in collective enterprises…through VCD” [12] (p. 494). This study responds to this gap by presenting women’s perceptions of gender-based constraints in relation to their work in a VCD collective enterprise.

Perhaps most notably, our study site of Touba, Senegal, not only offers an opportunity to gather data where rigid gender norms limit women’s participation in value chains, but it also allows for an opportunity to illuminate the perceptions of women’s empowerment where some may least expect women’s participation in a value chain to occur in Senegal. Despite being restricted in their mobility outside of the home in the religious city of Touba, as discussed in the next section, women perceive that their participation in the instant fortified flours value chain has led to improvements for themselves, their families, and communities. For instance, our study finds that women perceive that their VCD participation has led to the creation of a child care center and infrastructure in their communities that speak to their ideas of women’s empowerment. Given that women also perceive domestic conflict in relation to their VCD participation, our study further contributes to the discussion of the complexities of empowerment in the literature [49]. This study’s unique context of Touba, an important center that is explained in the next section, provides an interesting case study of women’s empowerment in a VCD project.

In sum, this study contributes to the scholarly literature and practitioner discussions on gender in agri-food value chains, women’s empowerment and nutrition, and gender equality and food security, by examining the perceived impacts of VCD on processors and mid-stream actors (retailers, farmers, and medical personnel) in a non-export-oriented agricultural value chain. Through a mixed-methods study that includes quantitative surveys, interviews, observations, and novel participatory activities, we find that the instant fortified flour value chain is not only perceived as helping improve women’s empowerment, income, and household nutrition, but medical personnel also perceive improved nutrition for their under or malnourished patients. Women processors and retailers are connected to a value chain through a woman-owned business, which received extrusion and fortification technologies from the VCD project, and this study’s findings speak to the complexities and multi-dimensionality of empowerment, as these women perceive gender-based constraints alongside women’s empowerment in their value chain participation. Specifically, women perceive that a significant barrier to their empowerment is their husband’s lack of understanding and the limitations placed on women’s mobility. The study site’s context, which is a socially conservative religious community, shows that while empowerment is possible through women’s participation in a food processing value chain, challenging rigid norms requires the inclusion and gender sensitization of other actors, such as religious leaders, in VCD projects, where rigid gender norms disempower women who participate in the value chains.

This article’s outline is described here. In the remainder of this Introduction section, we first provide a brief background/context of the study site. Then, we provide information on the VCD project’s intervention. The Introduction is followed by a Methods section that details the surveys, sampling design, participatory research activities, and additional data, such as the observations. Next, we present the Results of the study for each of the key stakeholders. We acknowledge the study’s limitations before moving into the Discussion section, which focuses on income, nutrition, and women’s empowerment in relation to the literature and the implications of the study. This is followed by the Conclusion.

### 1.1. Study Site Background/Context

We conducted our study in Touba, Senegal, located in an agricultural (peanuts and millet) and pastoral (zebu cattle and goats) area of the Senegalese Sahel. Touba is notably home to the powerful and influential Mouride (Muridiyya) brotherhood, a branch of Sufi Islam. Senegal’s population is approximately 94% Muslim, most of whom belong to one of the country’s four brotherhoods, which contributes to the stability of this West African democracy [50]. The Mouride brotherhood represents about 35% of Senegal’s Muslims and maintains close ties with the Senegalese government [51,52]. The Mourides’ leader, the Caliph-General, “is considered the country’s most influential public figure. Even the President kneels before him in public” [52] (p. 40). The Caliph-General and other Mouride elites have been considered “kingmakers” who can signal for whom their members should vote in elections [51] (p. 145) [53] (p. 74).

The Mourides are important to Senegal’s global trade network, and the brotherhood “controls the production of peanuts [the country’s main export crop], other tradeable products, and craft industries” [53] (p. 74). The Mourides’ “industry links the [capital] port of Dakar to an import-export economy based in” Touba, the seat of this powerful brotherhood [54] (p. 717). Touba is second only to Dakar in terms of its economic importance to Senegal [51] (p. 144). Touba is also a holy city and the most important pilgrimage site for the Mouride brotherhood. Each year, Touba hosts “Le grand Magal”, during which upwards of four million people come to the city to celebrate the life of the brotherhood’s founder, Cheikh Amadou Bamba [55].

Within the holy city of Touba, there are more rigid gender norms and socio-cultural constraints than in the rest of the country. While Koranic education is available in Touba, socio-cultural “factors limit girls’ attendance” and maintain “the exclusion of girls from school” [56] (p. 115–116). Many women who are born and raised in Touba have “had little to no formal education” and may disapprove of women or co-wives who are educated and want to work outside of the home [57] (p. 75). One study notes the more conservative approach to Islam in Touba, where a woman “had been publicly upbraided for wearing pants under her ankle-length dress, as pants are not permissible for women in Touba” [57] (p. 74). Beyond television, “other forms of amusement and escape are prohibited” in Touba [58] (p. 620).

Particularly relevant to our study in Touba is the pronounced lack of opportunities for women to earn their own incomes, especially outside of the home, and to provide for their families. Women must receive permission from their husbands to leave their homes, and in Evers Rosander’s study, women “mentioned the problem of husbands refusing to allow them to work outside the home and women traders have faced particularly strong opposition,” thus limiting which opportunities women can take to improve their own and their families’ wellbeing [59] (p. 166). As previously noted, women’s empowerment is a gender-sensitive VCD goal to improve gender equality as well as to reduce poverty across households. In the Diourbel region, where our study site is located, 47.8% of the population is under the poverty line, and 16.2% of the children display symptoms of malnutrition [60]. In addition, households in this agricultural and pastoral region earn a median annual income of 580,000 CFA (~983 USD) [61].

In sum, our study’s context of a religious city presents an interesting case study because, despite the rigid gender norms in Touba, women perceive their empowerment to come from their participation in an instant fortified flour value chain. Women also share that they feel disempowered, and our study captures their complex perceptions of empowerment related to their VCD participation. Finally, given the poverty and malnutrition in the Touba area, our study reveals the opportunities for income and nutrition improvements, as viewed by multiple key stakeholders in an instant fortified flour value chain: women processors and retailers, farmers, and medical personnel who are interested in the instant fortified flours for treating under and malnourished patients in Touba.

### 1.2. Intervention

The Touba Darou Salam Cereal Processing Unit (from here on, referred to as TDS) was created in 2015 in Touba. It was founded by an influential member of the Mouride community, Mme Mbacke, who was a favored wife of a Caliph-General. As previously noted, the Mouride brotherhood is a powerful religious sect in Senegal that is based in Touba. Mme Mbacke’s marriage to a Caliph-General places her in an elite position in this religious community, so she was a key contact for the project’s VCD goal to empower women in the region. Her business drew upon Senegalese women’s association networks called Groupement d’Intérêt Economique (GIE) or the Economic Interest Group (an organization with legal status that allows women to have easy access to credit through the mechanism of shared guarantees) to build the business and connect with 115 local women who could work as processors and retailers. TDS aims to address both malnutrition and women’s disempowerment in Touba by increasing access to affordable, locally produced, nutritious foods and improving household incomes through women’s employment. Aside from the nutritional gains for consumers, the sales of instant fortified flours could increase the participating women’s abilities to purchase nutritious foods as well as increase their ability to invest in education for the wellbeing of the family.

In 2016, a USAID-funded project partnered with TDS and provided capital in the form of an extruder, along with technical support and training. Each extruder can produce 35 kg of product per hour (max. production is 20 h a day or 700 kg/day). As a result of this partnership, TDS began producing an innovative product, instant fortified flours, using extrusion technology and fortification techniques. When mixed with water, instant fortified flours become porridge, which is a staple breakfast food in Senegal [62]. This product does not require cooking and is made with locally available millet and fortificants (moringa and baobab). Prior to the USAID-funded project’s involvement, fortified instant porridge was not available in Senegal. Additionally, TDS did not have the capacity to utilize the extrusion or fortification techniques.

This VCD project involved multiple types of upgrading. Economic upgrading occurs when firms move into higher value-added activities to gain further benefits from participating in production [63]. As a part of the project, the value chain experienced two types of upgrading: process upgrading and product upgrading. Process upgrading increases the ability of a firm to transform its inputs into outputs more efficiently by introducing better technology or reorganizing the production process [64]. TDS received an extruder as part of the project, which allows them to produce instant fortified flours far more rapidly than they could in the absence of the machine (the extruder blends and cooks the raw ingredients all in one step). Product upgrading can be described as moving into higher-value product lines [64]. Instant fortified flours were a novel, higher-value product (as compared to traditional porridge) that involved new extrusion and fortification techniques. Regarding the fortification of flour with local ingredients, the project set the iron, zinc, and pro-vitamin A levels so that they met 20% (minimum) of the daily requirements per serving size, and the team conducted the fortification analyses at reputable laboratories. The team includes local and international nutrition experts, such as Dr. Mario Ferruzzi, the director of the USDA Children’s Nutrition Research Center at Arkansas Children’s Hospital and professor and chief of the Developmental Nutrition Section of the Department of Pediatrics at the University of Arkansas for Medical Sciences College of Medicine [65]. The fortification entails a price premium with respect to normal flour, as established in a willingness-to-pay study [62].

## 2. Methods

**Figure 1:**
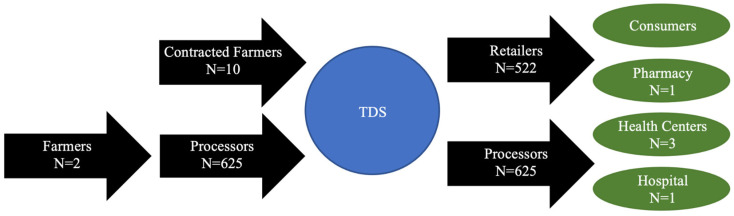
Instant Fortified Flour Value Chain.

The instant fortified flour value chain is composed of several key stakeholders, including small and medium-scale farmers, processors/retailers, and medical personnel, as illustrated in Figure 1. Figure 1 shows the participants’ locations in the instant fortified flour value chain, moving left to right from production to consumption. The far left represents the production phase of the value chain. Then, the processing phase of the value chain included: contracted farmers (contracted for the women retailers network) delivering raw ingredients to TDS and women processors bringing raw ingredients (that they purchase from farmers) to TDS, which receive the extrusion technology and fortification techniques from the project. Next, women retailers and processors sell the instant fortified flours to consumers and some medical personnel, who distribute the flours to under or malnourished patients. To interview these key stakeholders along the value chain, in January 2020, we visited: TDS’s processing facility; a point of sale for the flours; and medical facilities. We documented the impact of the project by surveying/interviewing women’s groups (i.e., leaders and randomly selected members from the groups) who are processors and retailers of the flours, farmers (who sell millet to TDS), and medical personnel who represented the health centers and a pharmacy and hospital that use and/or sell the flours. We also interviewed a private medical center that was interested in selling the flour to patients and interviewed another woman leader, Mme Bousso, who distributes much of the flour. In addition, we made observations and held participatory focus group research activities with 112 randomly selected women retailing and processing entrepreneurs to understand the impact their involvement has had on their empowerment, incomes, and households, as well as ways to improve the project moving forward.

In this 2020 study, we aimed to analyze the perceived impacts of value chain development on various stakeholders along the instant fortified flour value chain. We surveyed key stakeholders—including farmers (all men), processors (all women), retailers (all women), and medical personnel (men and women)—and held participatory focus group activities with approximately 120 women retailing and processing entrepreneurs to understand the impact their involvement with this business has had on their incomes, nutrition (specifically, household food security and dietary diversity), and empowerment. Our participatory activity questions for the women processors and retailers focused on their perceptions of: (1) how their lives have differed from before and after their processing/retailing, (2) what resources they use and need to improve their processing/retailing, (3) what barriers hinder their success, and (4) what do they value most about their involvement with the business. Our research questions for the farmers included the project impacts on their economic wellbeing. Finally, our research questions for the medical personnel focused on how and to what extent they utilize the instant fortified flours in their practices, as well as their perceptions of food security and nutrition outcomes.

Prior to the 2020 study, the project conducted focus group activities with women project participants in 2016 and 2019, with the initial findings indicating positive empowerment outcomes for women processors and retailers. However, the participants were not randomized, nor were there survey components; thus, we returned in 2020 to conduct a more scientifically rigorous study of the women’s views of their participation in the value chain. Additionally, the 2016 focus groups took place not long after implementation, so our study also assesses whether the initially promising perceptions persisted over time. The 2020 study and all the prior gender research for this project have gone through an Institutional Review Board (IRB) approval for human subjects research.

### 2.1. Surveys and Sampling Design

**Figure 2:**
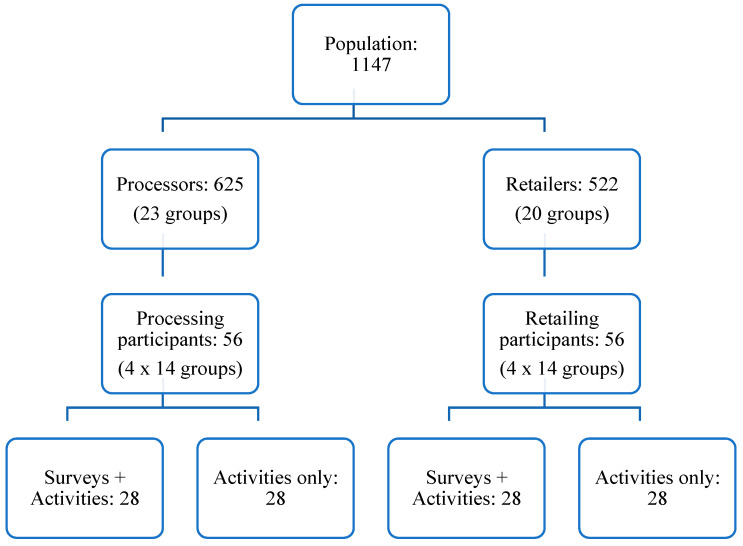
Sampling Design.

We interviewed various stakeholders along the value chain to assess the project’s wholistic impact on who TDS directly employs and on the community at large. On the production side, we conducted a quantitative survey with all ten farmers directly contracted by TDS and two farmers who sell grain to the women’s processing groups. These surveys focused on the demographics, millet production (including how much they sell to TDS), and financial inclusion. We also conducted quantitative surveys and interviewed the heads of health clinics, as well as a pharmacy and a hospital that use or distribute the flours. These surveys focused on how and when they became connected with TDS, how these facilities use the instant flours, as well as data on how much instant flours they used on a monthly basis. We also made space for these individuals to share their overall experience with the instant flours with us. Finally, we conducted quantitative surveys and held participatory activities, which are discussed in the following section, with randomly selected women processors and retailers to ensure we had a representative sample. For the survey, we collected data on the demographics, instant flour sales volumes, changes in household spending since working with TDS, length of involvement with TDS, and financial inclusion. We also took notes before/during/after our survey interviews so that the participants could elaborate on questions and provide ideas or thoughts on their participation in the instant fortified flour value chain. The sampling design of our surveys and the participatory activities are summarized in Figure 2.

We separately surveyed all 23 processing and 20 retailing group leaders and asked them to notify their members that we may be calling them in a few days. Then, we obtained lists of names and phone numbers (from each group leader) of all the women involved with the instant fortified flours. If a member did not have a phone, we obtained details on how to reach them through either another member, or as a last resort, the group leader. Then, we randomly selected 14 groups of processors and 14 groups of retailers. From these groups, we randomly selected four women for the participatory research activities, which are discussed in the subsequent section. If a participant did not pick up the phone, we waited 5 min, and if they did not call back, we contacted the next participant on the randomly ordered list. Of the four women selected from each group, we randomly selected two of those members to be surveyed prior to participating in the activities. These individuals were told to arrive earlier in the morning than those who were only participants in the activities. In total, we had a sample size of 112 women from 14 processing groups and 14 retailing groups for the participatory activities, 56 of whom were to be surveyed.

All surveys were conducted with the assistance of a tablet and were designed and programmed in the World Bank’s Survey Solutions™ software. Each stakeholder group had its own survey. The surveys were written in English, and upon arrival, we adapted the questions and response options to the local context. Trained surveyors interviewed the participants in either Wolof or French.

### 2.2. Participatory Research Activities

The purpose of the participatory activities was to understand how the women processors and retailers perceive any impacts of the project-supported business in their lives and to give them a chance to openly share their experiences with each other (and generate community discussion). Trained women facilitators spoke in Wolof and French. The participatory research activities provided a non-threatening way to discuss sensitive topics (e.g., gender roles, domestic conflict, etc.), did not require literacy skills (to better include diverse groups of women), and allowed us to get at the values that are hard to gather in a survey. In each activity, the participants worked in small groups of 3–4 women before sharing what they discussed with the whole focus group.

We conducted three different participatory activities during the January 2020 focus groups of women processors and retailers. The first activity was called “Before and Now.” Diagrams were used with the goal of understanding how the project’s support of TDS has affected their lives. For this activity, the women were asked to think back about their individual lives before becoming involved with the processing/retailing of instant fortified flours. The women were then asked to think about how their individual lives changed because of their own processing/retailing of the instant fortified flours. In small groups, the participants discussed how their individual lives differed from before and after their processing/retailing of the instant fortified flours. The participants were then asked to draw and/or write how they viewed their lives “before” and “after” their involvement with instant fortified flours.

The second activity, called “Resource Mapping”, had a goal of discovering what resources the women use, could use and need (to improve their processing/retailing of the instant fortified flours and the project moving forward), and what barriers hinder their success. In the small groups, the participants drew four squares and then, within the four squares, drew and/or wrote responses to these four questions: “What do you use to be successful processors/retailers?”, “What don’t you use, but could use to be a more successful processor/retailer (i.e., they have access to it, but don’t use it)”, “What do you need to be a more successful processor/retailer?”, and “What are the barriers to being a more successful processor/retailer”.

The third and final activity was called “Pie Charts”, whose goal was to learn what the women value most about their involvement with selling instant flours. In their small groups, the participants discussed, “What’s most important to be a successful distributor?” They then drew a circle and divided it into different sized pieces, and then assigned each of their answers to a different sized portion, based on how important it was (most important gets the largest piece, followed by second of importance; third of importance; and fourth of importance). Since this activity was the simplest, we chose it as the last activity, since we knew the participants would be more likely fatigued at this point in the day.

### 2.3. Additional Data/Activities

In addition to the surveys and participatory focus group activities, we interviewed a private medical doctor and met with key government leaders of Touba and the Senegalese government’s anti-malnutrition agency (Cellule de Lutte Contre la Malnutrition or CLM) to discuss the impact of the instant fortified flours value chain. During these meetings, we noted observations in meetings with women processors and retailers. We also interviewed the heads of a few clinics who do not currently utilize the flours but are interested in doing so, and the heads of a few health mutuals who offer reimbursement to clients who purchase the flours. These are all discussed in the next section, where applicable to the results. (We also note that this study complements the additional focus group discussions held in 2019 in Touba, Senegal, to assess women’s empowerment in processing with this business).

## 3. Results

### 3.1. Stakeholder Interviews

#### 3.1.1. Women’s Surveys

Through our surveys, we learned that local market demand for instant fortified flours is high, and women retailers can easily sell the product in local markets. We found that the number of Senegalese women working as processors and retailers for TDS has grown nearly tenfold from 115 at the project’s inception in 2016 to 1147 in 2020. Between January and September 2019, TDS produced nearly 140 tons of the instant fortified flour, which has a market value of USD 287,190 (each kilogram of instant flour sells for CFA 1250 or USD 2.06). Based on the first three quarters of the production data provided by TDS, TDS was on track to produce 186.5 tons of instant fortified flours by the end of 2019, representing a 4% increase in output since 2018, and a 7.2% increase in output since 2017.

Table 1 provides descriptive statistics of the study’s women processors and retailers. The average age of the women involved in either the processing groups or the retailing groups was around 42 years old (Table 1). These women often had little formal education (2.95 years). (There are 10 fewer observations for education due to changes in the survey form after the first day of our data collection.) Most identified as members of a religious group (86%) and a social group (93%), and some also identified as members of a political group (20%).

**Figure 3:**
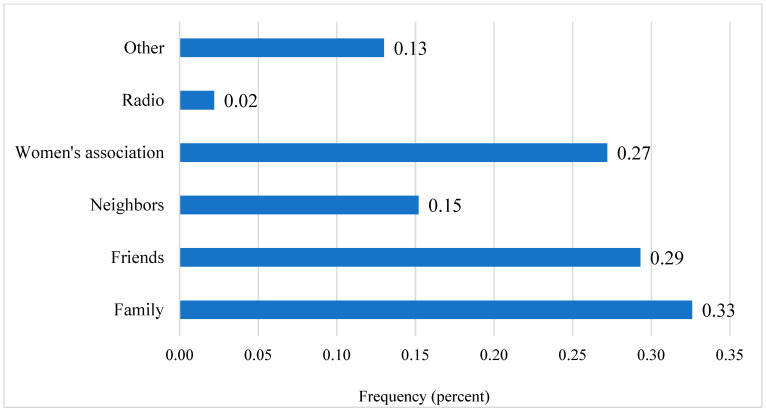
How Women Found Out About Processing or Retailing Job.

Figure 3 shows that most women found out about this job opportunity through friends or family (29% and 33%, respectively). Many also found out through their women’s association (27%) or from neighbors (15%). Survey responses for the non-leaders of the women’s retailer and processor groups indicated that 17% of the non-leader retailers and 30% of the non-leader processors were already connected with CLM, the Senegalese government’s anti-malnutrition agency, whose role we explain elsewhere in this article.

**Figure 4:**
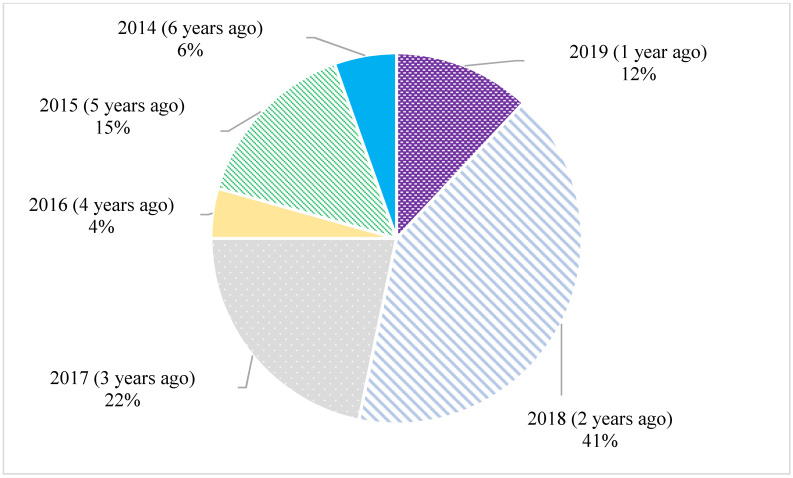
Processors and Retailers: Year They Started Processing or Retailing.

Figure 4 shows that most women became involved with the project in 2018 (41%), and the vast majority (75%) became involved from 2017–2019.

During a typical month of instant flour sales, the processors sell a median of 40 kg, providing $122.19 in revenue. (At the time of this study, the exchange rate was CFA 605 to USD). The flours are not always available, since the current demand outstrips supply; thus, this is what each processor is making in revenue for those months that they do have flour to sell. The retailers tend to sell 25% more flour than the processors (50 kg total), taking in USD 126.12 during a typical month of sales. For the retailers, since we know the price they purchase the flour for and the price they sell it for, we found that they make about USD 43.48 in net revenue [(sale price- purchase price) × median sales] during a typical month.

**Figure 5:**
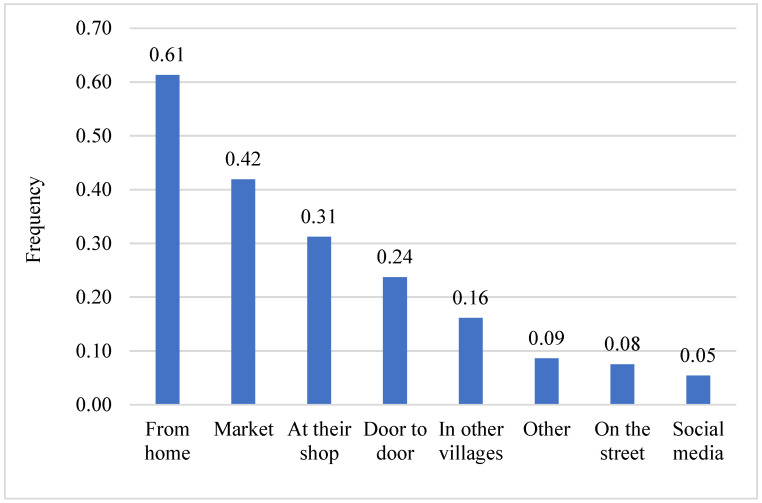
Locations Where Women Sell Instant Flours.

Figure 5 shows where the women retailers sell the instant flours. The women sell the flours at various locations; however, most frequently, the women reported selling from their homes (61%) and at the market (42%). Some even sell the flours through social media, such as WhatsApp or Facebook (5%).

**Figure 6:**
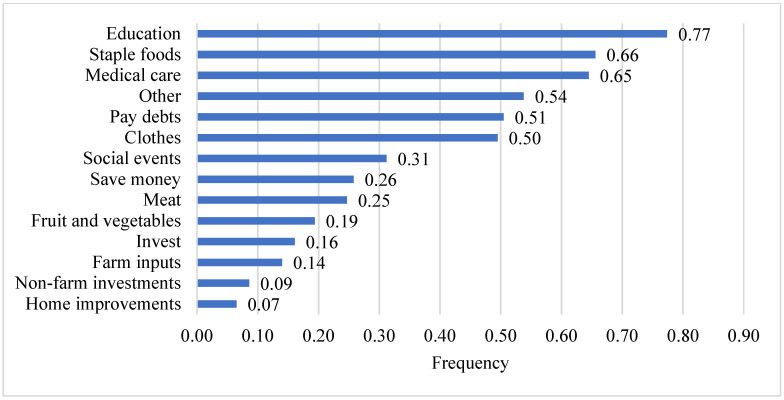
Reported Increases in Spending.

Ninety-eight percent (98%) of the women surveyed reported their household income increased due to their involvement in the program. Figure 6 shows how they spent this increased income. Most notably, 77% reported increased spending on education for their children or themselves. Others increased their spending on staple foods (66%), medical care (65%), and paying off debts (51%). Nineteen percent (19%) reported spending more on fruits and vegetables, and 25% reported spending more on meat, increasing their dietary diversity.

#### 3.1.2. Farmer Surveys

Table 2 provides descriptive statistics of the study’s farmers (ten contracted and two non-contracted farmers). The farmers selling grain to either TDS or individual women’s groups tended to be highly experienced (33.2 years) and have large operations. On average, these farmers cultivated nearly 40 hectares each, which is far more than a typical smallholder farmer in the region. Additionally, these farmers are harvesting large quantities of millet (29.1 tons) and are selling far more (125.6 tons). This indicates that these farmers are serving as aggregators by purchasing millet from other farmers and then re-selling it at a profit. As a result, their average revenue, just from millet, is over USD 50,000. All ten contracted farmers reported that their income had increased due to the contract, which had become more consistent and predictable. One farmer indicated that he receives a better price than at the market, and two farmers indicated that they have an increased ability to save and handle unexpected expenses.

#### 3.1.3. Medical Personnel Surveys and Interviews

Overall, our interviews with the medical professionals revealed a reported growing demand for instant fortified flours to combat community malnutrition. The Matlaboul Fawzaini Hospital and its associated pharmacy started purchasing bags of instant fortified flour from TDS in 2017 to send home with malnourished patients. The hospital and pharmacy currently purchase a total of 800 kg of flour every month but expressed a desire to increase their purchases by 20 percent (to one ton). Of their total 800 kg monthly purchase, 500 kg goes to the hospital, where it is given to children, and 300 kg goes to the pharmacy, where it is given to women, the elderly, and children.

A few public health clinics in Touba purchase the instant fortified flour for resale. On average, the clinics report that they purchase 390 kg monthly to resell to patients; however, they report that this amount doubles in the lean (hungry) season. In exceptional cases, the clinics give small bags of the flour away for free to malnourished children, with a nurse telling us that this is because malnourished “children cannot wait”. Community members can come to the clinics for nutritional assessments. Additionally, these clinics conduct a community nutrition outreach program to inform households about malnutrition and instant fortified flour. Trained teams of women go door-to-door in the local area to document the nutritional progress of those patients who have previously visited the clinic, as well as provide outreach to other community members who appear to be malnourished or have malnourished household members. Unlike the hospital and pharmacy, the clinics also feed the instant fortified flour to patients who must stay overnight.

**Figure 7:**
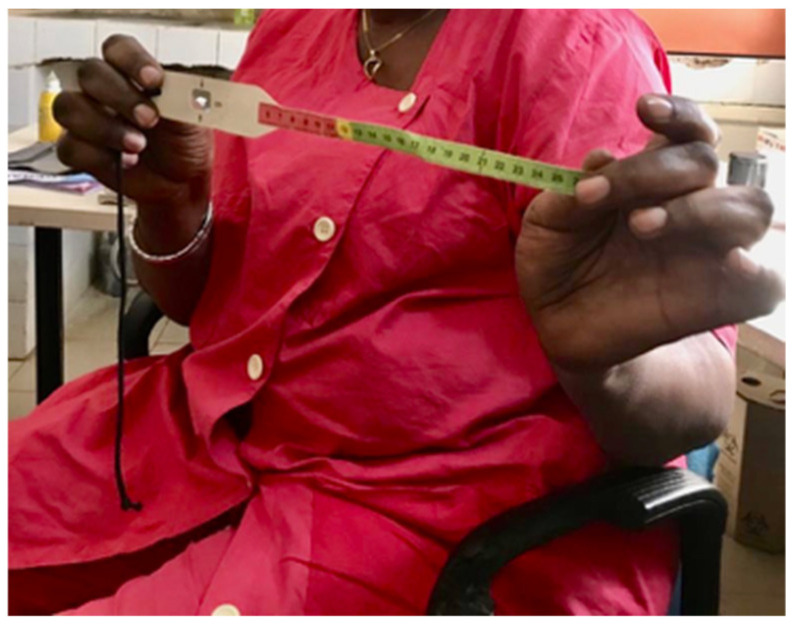
Nurse showing a child malnutrition assessment device.

One of the clinics we visited was run by a woman nurse who also runs two other clinics in the poorest areas of Touba. She was the newly elected head of Touba’s 31 public health clinics and wanted all the local clinics to use the instant fortified flours because she believed that the flours had had very positive impacts on her patients. During our visit, she showed us a device (Figure 7) that goes around the arm of a child to assess their nutritional status. She said that children “sometimes go right from the red to the green [in a couple of months] because of the flours”. She explained that red is dangerous and that children show nutritional progress as they move to green. She attributed the nutritional progress, however quick or slow, to all of her child patients with their intake of the instant fortified flours. We also met with the head of a health mutual, which is the local equivalent of health insurance. The mutual does not sell or give away the flours, but it reimburses 80% of the purchase price for its members. The head of the health mutual views the instant fortified flours as part of the prevention and treatment of malnutrition.

### 3.2. Participatory Research Activities

#### 3.2.1. “Before and After” Activity Results

**Figure 8:**
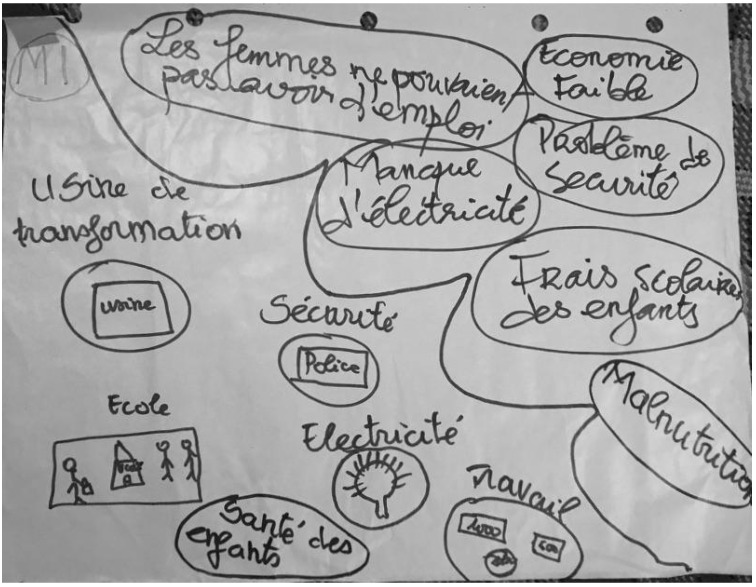
Example of “Before and After” Activity with Processors.

Figure 8 shows an example of a “Before and After” activity with one of the women processor groups. As a reminder, this activity focused on the changes that the participants perceived in their lives because of their involvement with the instant fortified flours project. An overwhelming response from the participants pointed to gaining independence, a new sense of community and camaraderie, and an increased knowledge of agriculture and other skills. The women processors and distributors said that, after joining their respective women’s groups, many positive changes occurred in their lives, households, and communities. Specifically, we can group these changes into three main categories: gender relations, household finances, and education. The participants told us they saw improved (attitudes toward) gender equality and women’s empowerment. They felt more respected by their husbands and community members because they were earning their own income and could help provide for their household needs. With this increase in income, women felt that their households’ financial, health, and living conditions improved. For example, some women perceived that their involvement in the project led to improved infrastructure, such as access to water in their communities. They reported an increased ability to pay for their medical needs, electricity, houses, and home furnishings. Additionally, they reported being able to invest more in education for their children, from elementary through to university, and for themselves by taking classes. They also learned various skills through project and business training.

#### 3.2.2. Resource Mapping Activity Results

**Figure 9:**
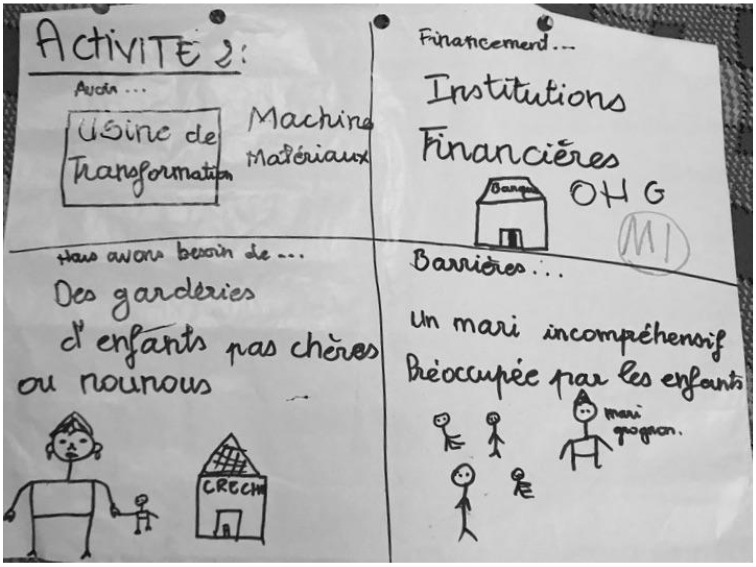
Example from Resource Mapping Activity with Processors.

Figure 9 presents an example of a resource mapping activity with one of the women processor groups. This activity involved four questions that focused on the processors’ and retailers’ perceived access to resources and the perceived barriers to their success.

The first question that we asked each group focused on their views of what helps them to be successful processors and retailers. The participants noted the importance of their social funds, also called a solidarity box or “caisse de solidarité”, which each women’s group has and to which women contribute money to support fellow members in times of need. They also shared that their relationships with each other help them to be more successful, as well as their good salaries earned from their instant fortified flour activities.

Next, we asked each focus group about underutilized resources, which are things that they do not use but have access to and could use to become more successful. The participants discussed that they have access to the following but that they could use these resources more to be successful: training; school; help/support; credit; agricultural bank; financing partnership; NGO partnership; financial help; and machines for packaging and for millet. These underutilized resources represent a summary of what all the groups presented. Thus, it is possible that some women did not perceive that they, as individuals, have access to each of these resources.

Thirdly, we asked each focus group about the things they need to become more successful but that they do not currently have access to. The participants felt they would greatly benefit from having more training or schooling related specifically to marketing, agriculture, and computer skills, particularly Microsoft Excel, for bookkeeping. They also noted that they would be more successful if every woman had a cell phone for improved communication. Some participants noted that they need better internet access or would benefit from additional partnerships with more (agricultural) banks, NGOs, and the government. The women also expressed the need for a childcare center that is affordable and safe. Some expressed that it is not safe to leave their children at home when they go to work because the children are at risk of kidnapping or rape if left alone. Others felt that they needed a greater “freedom to work” due to a lack of support from their spouses, who did not understand why they needed to leave home and work.

Finally, each focus group discussed their perceived barriers to being more successful processors and retailers. By far, the most cited barriers across the focus groups were their husbands’ lack of understanding or support and the rigid gender norms regarding travel outside the home. The women discussed how their husbands become jealous of their activities and make accusations of infidelity, and sometimes respond with domestic violence. The wives of overseas migrants noted that since their husbands are not around, their husbands have more trouble understanding or trusting the project and fear their wives working. The women’s abilities to travel and the distances they can go depend on their husband’s consent, as explained by the focus groups on our study site. One participant summarized the focus groups’ consensus on why this barrier is so strong in this community: “Religion [is a barrier to us being more successful] because for Muslims in Touba, women must ask the husband for permission to do anything, and religion because of the limited distance that women can work from home”. As echoed in the previous prompt, time constraints with children and lack of childcare are also reported barriers to their success. “Many women do not have family planning and give successive births. Many women give birth repeatedly,” explained one participant. Other barriers to being successful included: time constraints from household chores (e.g., cooking, fetching fuelwood), the lack of transportation or access to a car, and the jealously of other wives in polygamous households.

#### 3.2.3. Pie Chart Activity Results

**Figure 10:**
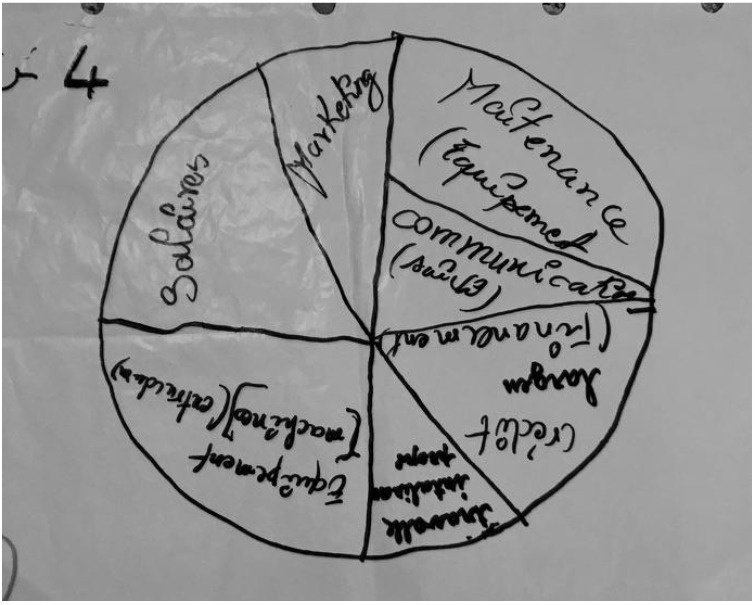
Example from Pie Chart Activity with Retailers.

Figure 10 shows an example from a pie chart activity with one of the women retailer groups. For this activity, the focus groups ranked the importance of their responses to this question: “What’s most important to be a successful retailer/processor?” Overall, the focus groups typically ranked the following as the most important for their success: marketing, gaining experience, equipment, good salaries, solidarity box (social fund), having qualified staff, partnerships with farmers, access to storage facilities, and the freedom to work (as women). For their second list of importance, the groups listed maintenance, accountant, financing/financial advice, car (for transportation), customers, and respect for each other. A few groups ranked some of these lower than the most important or of secondary importance. The third ranking of importance included: access to credit (financing for selling), NGO/government/private partnerships, electricity, and water. Finally, the women listed the following as fourth in importance for their success: visibility of their business in the community, saving money, having an employer with a high level of experience, legal advice, and worker safety.

### 3.3. Additional Observations from Participatory Activities

#### 3.3.1. Polygamous Household Dynamics

In polygamous households in our study site, the wives share cooking duties on different days. In our study, some women processors said they want an assistant to help with cooking because the co-wives become upset if one of the wives leaves to work on a day when she is supposed to cook. This need to return home in time for cooking in a polygamous household was observed in one group, as one participant was insistent that she had to leave early to cook due to the tensions in her polygamous household. She was visibly nervous that she might be late to start cooking. The other women in the group agreed that there would be a domestic conflict if she did not leave early to cook. During the focus group discussions, we also documented women’s comments that there is often jealousy in polygamous households if one wife leaves to work and the other wives must stay at home.

#### 3.3.2. Mobile Phone Access

The group leaders gave us phone numbers to contact women processors/distributors, and we observed that to reach various women, we had to go through their husband’s cell phone. In at least one case, when our staff called a woman distributor who was randomly selected, the husband kept telling the staff person to call back three different times because he was in the field or doing other things. This suggests that some women are very limited in their phone communications, including to be contacted for the project, for their women’s association, and in their daily lives. This limitation was also noted by multiple women participants in our focus group activities.

### 3.4. Results of Additional Data/Activities

#### 3.4.1. Private Clinic Interview

The head doctor of a private health clinic in Touba, Senegal, told us, “We welcome the project not only nutrition-wise, but because the products you are developing are made from our local vegetables, fruits, etcetera, the project is very welcomed here”. Stressing that there is a strong interest in instant fortified flour by the greater medical community, he said, “The Head of the Medical District of Touba, the Association of Medical Doctors of Senegal, and I have been assessing the product. We and the Association of Medical Doctors of Senegal [i.e., doctors throughout Senegal] want to use this product for the malnourished”. He wanted more information/proof of the product’s quality, safety, and availability and was most interested in whether it could be used for children under the age of five.

#### 3.4.2. Senegalese Government Involvement via CLM

Mme Sokhna Bousso Mbacke (another influential woman of the Mouride branch of Sufi Islam in Touba) partners with TDS as a retailer of the instant fortified flours and collaborates with the Senegalese government’s anti-malnutrition agency, Cellule de Lutte Contre la Malnutrition (CLM), to distribute the instant fortified flours across 100 sites. The government’s positive assessment of the instant fortified flours impact on combatting malnutrition has led to a pilot project between TDS and CLM. In the January 2020 interviews, both Mme Sokhna Bousso Mbacke and a CLM representative told us about the forthcoming pilot project that will draw upon the women’s processing and distributing network, which our study’s project has been supporting through TDS. CLM purchases/subsidizes 40 tons of the instant fortified flours for food security efforts, but the CLM representative stressed that CLM (the government agency) is looking to greatly increase their purchases/subsidies. If the pilot project proves successful, the government plans to adopt the TDS business model nationwide to increase the supply of instant fortified flours through women processors and retailers.

### 3.5. Limitations

Aside from not establishing causality, the limitations of this study include sampling from women retailers and processors to whom we could contact through cell phones, including through their husbands. Furthermore, perhaps the poorest women in the groups were not contacted due to no cell phone in the household, but each woman from these groups had a phone number associated with them (e.g., a neighbor’s phone number). There may have been some recall biases in the women’s recollections of their sales patterns or data, and our study was a snapshot of one point in time from their perceptions. During the participatory activities, some women were more reluctant to share and seemed less comfortable with drawing or writing. The facilitators helped to improve the group dynamics where we discerned discomfort; only women were present during the participatory activities. For the survey portion of our study, we could not ensure a female surveyor for each survey participant; we had two female surveyors and two male surveyors, so some women survey respondents may have replied differently to the male surveyors than the female surveyors.

## 4. Discussion

In this section, we first discuss the perceived impacts of the VCD project on income. Then, we discuss the key stakeholders’ perceived impacts on nutrition. Finally, we discuss the perceived impacts on women’s empowerment. The implications of the study and its findings are raised in each sub-section topic (income, nutrition, and women’s empowerment) in relation to the literature.

### 4.1. Perceived Impact of VCD Project for Key Stakeholders: Income

Men farmers, women processors, and women retailers are the key stakeholders for whom data was collected on their perceived income changes since their involvement in the VCD project. The survey findings reveal that the instant fortified flour value chain is an income-generation opportunity for both men and women. While the men farmers harvest their own millet, they also serve as aggregators by purchasing millet from other farmers and then re-selling it at a profit, with their average revenue from millet reportedly reaching just over USD 50,000. The woman-owned TDS business has contracts with ten farmers who perceive that their participation in this value chain has increased their income. Although one farmer reports that he could obtain a better price at the market than at TDS, the ten farmers perceived that their contract with TDS had made their income more consistent and predictable. Two farmers even reported that their participation in the project’s instant fortified flour value chain had increased their ability to save and handle unexpected expenses. This value chain appears to be more lucrative for the men farmers than the women processors and retailers based on the surveys and, as discussed below, but many more women than men are employed through the project-supported TDS business.

Women’s labor force participation and income are two indicators of women’s financial inclusion in a value chain. Based on data supplied by TDS, the number of Senegalese women working as processors and retailers for TDS has grown nearly tenfold, from 115 at the project’s inception in 2016 to 1147 in 2020. The vast majority (75%) of these women joined from 2017 to 2019. During a typical month of instant fortified flour sales, women processors reported USD 122.19 in revenue and women retailers, who typically sell 25% more than the processors, bring home USD 126.12. Notably, this is the revenue for months when they have flour to sell because they report that the current demand outstrips supply. If their perception of a high local market demand for instant fortified flours is correct, the project could be expanded to increase the supply, thereby increasing these women’s potential earnings from their instant fortified flour sales. Similar to the farmers’ positive views of income generation due to their involvement in the instant fortified flours value chain, ninety-eight percent (98%) of the women processors and retailers surveyed perceive that their household income has increased due to their involvement in this value chain. Further, in the participatory research activities, the women processors and retailers echoed this belief by listing their “good salaries” as a positive change that has occurred since they became involved in the instant fortified flours value chain.

Women’s reported income generation in the instant fortified flour value chain is important because economic empowerment is heralded as one pathway for women to work toward achieving their full potential and advancing women’s rights [66]. However, the women’s positive perceptions of earning an income do not illuminate the women’s actual control over how their income is spent. Women’s economic empowerment (WEE) includes women’s influence over household expenditures, and this study did not capture the complexities of such household decision-making. In addition, even if women report or perceive some economic empowerment through income generation, women may also feel disempowered by the added time constraints of employment, especially if their household work is neither shared nor decreased once employed. Indeed, we find that women in polygamous households can come into conflict with co-wives who are not employed in the project because there is jealousy as well as pressure to rotate the cooking responsibilities in the household. In this sense, there is some disempowerment or at least constraints facing women in polygamous households due to housework obligations. We discuss more examples of disempowerment for women in the value chain further below.

While capturing the full complexity of WEE goes beyond the scope of this study, and scholars and practitioners continue grappling with measuring WEE [67], it is still valuable to hear from women processors and retailers that they share positive perceptions about their income earned through participation in the project’s instant fortified flours value chain. The VCD project, with donor support, could build upon this finding to evaluate the project and the value chain’s continuing influence on the advancement of gender equality and women’s empowerment through a wider array of WEE-related measures, such as those released in a November 2020 report titled *Measuring Women’s Economic Empowerment: A Compendium of Selected Tools* [68]. In addition, an impact evaluation of women’s empowerment through the instant fortified flours value chain may benefit from the lessons learned by the Women’s Economic Empowerment (WEE) Measurement Learning Collaborative, launched in May 2021 by the Center for Global Development (CGD) and Data2X, a gender data alliance housed at the United Nations Foundation. Given that the Touba, Senegal instant fortified flours project is expected to be modeled across Senegal by the Senegalese government, as discussed further below, donor support for a larger-scale and more comprehensive study of WEE could create an opportunity for subnational comparative WEE research, as women’s processing and retailing networks throughout Senegal may join an instant fortified flours value chain. It would be particularly interesting to compare the complexity of WEE in the subnational areas beyond Touba, a very conservative religious area of Senegal, to assess the in-country variation in women’s empowerment opportunities and barriers.

### 4.2. Perceived Impact of VCD Project for Key Stakeholders: Nutrition

The VCD project’s value chain is nutrition-sensitive in that the processed flours are fortified, and value chains with nutrition-sensitive interventions have the potential to help reduce malnutrition and increase (demand for) the consumption of nutritious foods [7]. In this section, we discuss the VCD project’s nutrition benefits as perceived by these key stakeholders in the instant fortified flours value chain: women processors, women retailers, and medical personnel. We first discuss the perceptions of the medical personnel and then those of the women processors and retailers.

In the surveys and interviews, the medical personnel reported demands by the medical community for more instant fortified flours from the TDS business. This is tied to their perceptions that the instant fortified flours could or do help to combat malnourishment. Perhaps most notably, the head of Touba’s 31 public health clinics wants all the local clinics to use the instant fortified flours because she perceives the flours as having had a positive impact on malnourished patients that come to her clinic. She explained that she had observed the positive health impacts of the consumption of the flours by her use of a child malnutrition assessment device (as shown earlier in a photo), which at times had moved her patients from being dangerously malnourished to appearing to be nourished. This reported observation from the head of public health clinics in poor neighborhoods of Touba suggests that the instant fortified flours should be studied in the field to confirm these observations and go beyond the positive laboratory findings of the nutrient-fortified flours. For instance, a biomedical study in Touba could draw upon the aforementioned nurse outreach program to target malnourished populations and assess their response to the VCD project’s instant fortified flours, which are conveniently processed in Touba. Such a study would also supply the Association of Medical Doctors of Senegal, which has been assessing the instant fortified flours according to interviews, with more data to ensure proof of the product’s quality and safety for patients of various ages. Currently, the flours are being given to women, children, and the elderly by medical personnel, in accordance with the surveys and interviews with private and public medical personnel.

The availability of the instant fortified flours could be increased through the Senegalese governmental anti-malnutrition agency’s plans to expand the value chain throughout Senegal. Such an expansion would open up opportunities to increase women’s participation in the value chain throughout Senegal. Currently, the government’s agency, CLM, will distribute the instant fortified flours across 100 sites in their pilot study to assess the flours’ impact on combating malnutrition. This pilot project between TDS and CLM provides income to the woman retailer who contracts with the government. This woman retailer has a high social status in Touba due to her religious ties via marriage. Long term, if the value chain is expanded throughout Senegal, it would be interesting to involve more lower-income women in the more lucrative positions in the value chain, such as by training and creating opportunities for lower-status women to be owners or co-owners of a business, or to have contracts with large buyers, such as the government, for a particular vicinity. A larger study could then compare the impact of the value chain on women from diverse economic and social backgrounds in Senegal. Touba, which of course, is unique in its hierarchical religious relations, and so tapping into women of high status in Touba has been particularly helpful for the VCD project to begin and grow. Extending beyond Touba, it would be useful to ensure that women of diverse religious and social status backgrounds have equitable access to the more lucrative aspects of the value chain for women.

There is a potential to study different mechanisms or pathways to achieving improved nutrition by tapping into the Touba VCD project (as well as any future expansion of the value chain throughout Senegal). First, what is the relationship between nutrition and the consumption of instant fortified flours? Specifically, how are general consumers and patients impacted nutritionally by the consumption of instant fortified flours? Are the women processors/retailers or their household members consuming the flours, and if yes, what are the nutritional outcomes? Second, does an increase in income and/or women’s empowerment among women processors/retailers impact the nutritional outcomes of these women or their household members? Do women exercise agency in the purchasing of nutritious foods based on their learning about combating malnutrition with instant fortified flours? Does household decision-making about nutritious foods or dietary diversity shift after women become processors/retailers in the value chain? If so, how? Are men in the household sharing in the decision-making of the purchasing of nutritious foods? If yes, have men in the household learned about more nutritious foods and dietary diversity from their wives’ participation in the value chain? These are some of the questions that could be researched in a study about the possible mechanisms or pathways to improved household nutrition from the VCD project’s instant fortified flours value chain.

While our study does not show a definitive shift in women’s decision-making roles in purchasing foods for the household, our study finds that women perceive that their households have increased spending on dietary diversity and more nutritious foods since they became processors/retailers in the VCD project’s instant fortified flours value chain. As noted in the prior sub-section on income, WEE, or more simply, women’s empowerment, includes women’s influence over household expenditures, such as the purchasing of nutritious foods for their children and other household members. The relationship between women’s empowerment and nutrition, or women’s income and nutrition, is important for this nutrition-sensitive VCD project. This USAID-funded project’s intervention aims to not only support the processing of a nutritional product (i.e., instant fortified flours) but also to support women’s empowerment in the value chain. While our study does not test causality between women’s participation in the value chain and their household spending on foods, our study does document women’s perceptions of how their participation in this VCD project’s value chain has impacted their household expenditures, including food purchases.

In this study, the surveyed women processors/retailers reported that since their participation in the instant fortified flours value chain, they perceived that their increased income from the value chain has led to increased spending on various household expenditures. More specifically, related to nutrition and dietary diversity, 66% of the women reported increased spending on staple foods; 25% of the women reported increased spending on meat; 19% reported spending more on fruits and vegetables. These survey findings complement the participatory research activity findings in which women express that their income from processing/retailing has contributed to their increased household spending on foods, thereby contributing to their (perception of) increased food security and dietary diversity for their households.

Questions of causality and mechanisms remain. Was there a shift in household decision-making on purchasing foods because of the women’s participation in the instant fortified flour value chain? If yes, by what mechanisms has the shift occurred? Were women empowered in decision-making to spend more on foods for the nutritional needs of the household? As previously noted, a future study could explore the complex relationship between women’s empowerment in an instant fortified value chain and the nutritional impacts on household members. The literature highlights the complexity of nutrition in relation to women’s empowerment: the correlation between the nutrition outcomes and dimensions of women’s empowerment is complex; some dimensions of women’s empowerment may not correlate with improved household nutrition; local contexts matter for women’s empowerment and their mediating role in household nutrition and dietary diversity [69,70,71,72].

Our study’s findings speak to the need for more comprehensive research on women’s empowerment in the VCD project’s instant fortified flours value chain in relation to nutritional measures in households. It would be a contribution to researchers, practitioners, and policymakers to study whether the VCD project’s nutritionally fortified product improves not only local consumers’ access to instant fortified flours but also women participants’, meaning the purchasing and/or consumption of the flours by the women processors/retailers and their households. Are these women and their household members consuming the flours? Is the income from the women’s participation in the value chain impacting their own or their household members’ nutritional outcomes, such as dietary diversity? Is there any spillover effect of the nutritional education about the instant fortified flours provided to the women from the VCD project? The question remains whether this nutrition-sensitive value chain has increased women participants’ empowerment and if any such empowerment has shifted household decision-making on the purchasing of foods (and other expenditures). Even if spending on food has increased from women’s earned income in the value chain, have women increased their sense of agency or influence in the household or community? Again, this study does not prove that the women participating in the project have more income or buy more staple foods, meat, or other foods. In sum, we need more studies on the mechanisms or pathways to the nutritional benefits in the instant fortified flour value chain, as well as the relationship between women’s empowerment, women’s participation in the value chain, and nutrition.

### 4.3. Perceived Impact of VCD Project on Women’s Empowerment

Beyond income as a proxy of an aspect of women’s empowerment and beyond women’s perceptions of food purchases, it is important to consider what women processors/retailers in the VCD project say they need, want, or appreciate in relation to their work in the value chain. In other words, women’s subjectivities of their own sense of empowerment or disempowerment matter [73]. There is not one single measure or index that captures the nuances of women’s empowerment as a process across time and place [74]. Our study participants live in a very conservative religious area of Senegal called Touba. While patriarchal norms exist globally, there are subnational variations in levels of patriarchy within countries (e.g., [75,76]). Within Senegal, the context that women face in Touba is unique, given its hierarchical religious relationships and rigid gender norms. The women in our participatory research activities expressed that the religious interpretation in this region limits their mobility and freedom to work. Senegalese scholar Fatou Sow confirms how influential and resistant Touba’s norms are to gender equality; despite the 2002 constitutional reform that introduced gender equality terms and the 2010 parity law, in “the 2014 local elections, a movement in Touba, a religious city and seat of a powerful Muslim brotherhood, refused to comply with the requirement for parity on the electoral lists, and was never sanctioned, as required by law” [77] (p. 8). If the government follows through with its plans to expand the value chain (drawing on the women’s processor/retailer network and the TDS business model) throughout Senegal, then policymakers and researchers could benefit from a subnational study of women’s empowerment in relation to women’s networks and VCD. In addition to exploring the subnational context differences, such a study could also illuminate the differences across a broader diversity of Senegalese women. While women in Touba’s participatory research activities expressed that they appreciated “good salaries” and increased food purchases, based on their perceptions of changes since they became involved in the value chain, our study did not disaggregate the diversity of individual women’s aspirations from the participatory research activities, which included 56 women processors and 56 women retailers. For some women, such as widows or women whose husbands are not providing sufficient funds as a breadwinner, perhaps their involvement feels more like a necessity rather than a choice or a sense of agency. In sum, there is much research required to better understand the subjective dimensions of empowerment and the processes of women’s empowerment and disempowerment across different groups of women and different contexts.

Interestingly, our study highlights the need for access to contraception, as expressed by the women in the participatory research activities. While this study focuses on women’s perceptions, the women expressed valid reproductive needs that are understandable worldwide. Women in our study spoke up about the difficulty of not being able to space their pregnancies and the challenges of having children “back to back” without respite. One medical provider, during our interviews, stressed that the women’s ability to access contraception is important to prevent malnourishment in households; this medical provider said that women in Touba often seek contraception in secret because their husbands do not approve of contraception. This is also concerning for the possibility of domestic violence should a woman’s use of contraception without her husband’s permission be discovered by the husband. The lack of women’s empowerment in the use of contraception should also be a concern for nutrition-sensitive value chains if the prevention of malnourishment is viewed from a more nuanced gendered lens rather than a simple sex disaggregation across nutritional outcomes. The inclusion of men and community leaders in gender-sensitive VCD projects must also be considered, as men’s and community leaders’ attitudes toward women’s empowerment and women’s reproductive choices impact women’s use of contraception and women’s agency in other areas of public health importance [78,79,80,81].

Household relationships impact women’s sense of empowerment and disempowerment. The threat of domestic violence or conflict may inhibit some women from exercising their agency to make decisions to benefit themselves, their children, or others. As noted, in polygamous households, co-wives can come into conflict with the study’s women processors/retailers due to jealousy and the obligation to rotate cooking days for the household. Such diversity among women and across households should be considered in projects so that all women can equitably access (lucrative) value chains, especially those with gender-sensitive goals and VCD projects [15,82]. The women in our study suggested that they need help to find cooks to hire so that women can work outside of the home without upsetting the dynamics between co-wives as well as meeting the housework expectations of their husbands. In the participatory research activities, the women stressed that their husbands could be a significant barrier to their freedom to work outside of the home due to accusations of infidelity as well as the local religious gender norms that women must receive their husbands’ permission to travel certain distances and to work outside of the home. In this sense, work in the value chain can at times be disempowering for women who face such tensions in their household relationships. This barrier of the husband’s jealousy toward wives working outside of the home or the husband’s limitations on the wives’ mobility outside of the home (and in line with Touba’s religious norms) is not only found in Touba. These interrelated constraints of a lack of the husband’s support for women’s work, women’s lack of mobility, and the threat of domestic violence by disapproving husbands are found in other studies and locations, and they are also barriers to other gender equality issues that we have discussed, such as access to contraception to space the births of children (e.g., [83,84]).

The women in our participatory research activities expressed that they perceive a need for childcare centers so that they can work outside of the home without concern for who is supervising their children. The literature shows that although there are some benefits from affordable childcare for women’s employment, there is not a clear-cut improvement across all areas of women’s empowerment, which is more complex than being able to work outside of the home and find childcare [67,85]. Interestingly, some women in our participatory research activities spoke about their collective or individual achievements as including the creation of a childcare center in at least one community. This discussion came about when the women explained how their lives have changed since joining the VCD project’s value chain. More studies are needed to examine any such community changes that women perceive as having ties to their work in the VCD project, which draws upon a women’s network of processors/retailers. If there is a connection between the value chain and women’s empowerment in terms of collective or individual mobilization in their communities, this would be an important connection to understand by researchers, practitioners, and policymakers, as women’s collective mobilization speaks to not only women’s sense of empowerment but also women’s abilities to improve their communities in very important ways. For instance, the women also said that they had gained more respect from their husbands (albeit, this is contradictory in terms of the threats of domestic violence, as noted above) and more respect in their communities after becoming participants in the VCD project’s value chain. The women suggested that this respect has enabled them to create other community and infrastructure changes, such as access to water, electricity, roads, and the creation of both a police station (that women say provides security) and a health center in at least one neighborhood. Again, these are women’s perceptions in our study, and follow-up research would be needed to understand the processes behind these community and infrastructure changes. Nonetheless, the women in our study believe that they have gained a voice in saying what needs to be done in their local communities after they became involved in the VCD project’s value chain. If these beliefs are accurate, we would also need to study the mechanisms or pathways to these changes and their relationship to women’s empowerment. For example, we could ask: is it the women’s collective network or solidarity among women processors/retailers that explain these changes, or are there individual women who have become community leaders following their newfound respect as wage-earners? Have women gained speaking and negotiation skills from VCD project training or their work experiences, and has there been a spillover effect toward negotiating for community changes that women want? Regardless of the answers, women’s perceptions of self-efficacy on some positive changes, such as the community/infrastructure changes as well as household financial and educational changes (as noted in our Results section), are important subjectivities that speak to women’s empowerment in relation to the VCD project.

The VCD project (and the potential expansion of the instant fortified flours value chain in Senegal) presents an opportunity to conduct more research on women’s empowerment on both the individual level and the collective level, as the Senegalese women’s network of processors/retailers is a collective that could become mobilized for basic or more transformative changes. Already, the women collectively perceive that their solidarity box or social fund (that supports individual women in times of need) is something that helps them to be successful processors and retailers. They also noted that their respect for each other helps them to be successful. In our observations of the women in the participatory research activities and in the before/after times of the activities, we witnessed a sense of camaraderie and solidarity among the women, greeting each other and introducing themselves to newer members that they had not met. We observed them speaking to each other about their problems and their achievements as they perceived them. There is potential that the VCD project’s use of the women’s network may have some positive spillover effects that we cannot predict for households and communities. Beyond women’s labor force participation in the value chain and their access to instant fortified flours from the TDS business, which benefited from the project’s intervention, the VCD project could be a stepping stone for some women processors/retailers to gain a sense of self-efficacy and therefore move toward collective mobilization for basic community needs or even transformative changes, such as gender equality reforms. This is, of course, aspirational; however, a VCD project that ascribes to a mission of women’s empowerment and gender equality could aspire toward women’s collective empowerment in the long term. Naila Kabeer’s work on women’s empowerment and theories of change are particularly insightful when thinking about how development projects could aspire to long-term transformative changes [73,86] (see also [82] on transformative change).

Finally, why did the participatory research group activities appear to have seemingly incompatible data or contradictions in some of the results? For instance, women expressed feeling more respected by their husbands as well as facing limitations by their husbands. In this example, it is possible to feel more respected due to a change in one’s income but to not always be treated with respect. It is also possible that husbands may show the women more respect in response to income or perhaps only in the community, but then maintain the rigid gender norms that give a husband authority to discipline his wife and limit her mobility instead of treating her as an equal human being. Such seemingly incompatible findings have been found in other studies in which women are recognized as income earners yet still face patriarchal constraints from their husbands [87]. Aside from empowerment being a very complex, context-specific, and multi-dimensional (e.g., social, economic, political) concept that can include contradictions, it is also possible that some of the seemingly incompatible views of women in our study are due to the questions we asked and the women’s interpretations of those questions. It could also be that some of our participatory research activity methods (e.g., rankings) missed out on the differences among individuals due to group consensus, although we analyzed all the responses on the small group posters to assess our notes from the larger participatory discussions when summarizing the rankings. In other words, not all individual women within a participatory research group may have experienced domestic violence or conflict in their households; perhaps some women in small groups talked about women they know who would like to work outside of the home but cannot due to their husband or their religion’s limitations. Due to the sensitive nature of topics like domestic violence/conflict and contraception, and as explained in the Methods section, we chose participatory research activities to explore women’s perceptions of their empowerment (or lack thereof). Follow-up research in this specific context of Touba, Senegal, would be needed to better understand the seemingly incompatible data of some responses from the participatory research activities.

## 5. Conclusions

Gender-sensitive VCD projects have the potential to positively impact stakeholders along the entire value chain, even in communities with remarkably strict gender norms and marginalized populations. This mixed-methods study in Touba, a very conservative religious area of Senegal, included farmers and medical personnel from an instant fortified flours value chain as well as women processors and retailers so that we could hear from these beneficiaries about the VCD project’s perceived impacts on income, nutrition, and women’s empowerment. We found that male farmers enjoyed financial benefits from selling their millet to a woman-owned business which has been supported by the VCD project, and women processors and retailers discussed income benefits from their participation in the value chain. Both women and medical personnel perceived the nutritional benefits from the instant fortified flours value chain, with women discussing increased spending on foods from their incomes as processors/retailers and medical personnel perceiving positive changes for their malnourished patients who consume the fortified flours. While these positive perceptions are promising, more research is needed to test for causal relationships reported by this study’s key stakeholders.

This study’s VCD project aims to support women’s empowerment through women’s participation in an instant fortified flour value chain. Yet, while women’s participation has increased since the start of the project, women’s labor force participation and income do not capture women’s empowerment, a complex and, at times, contradictory process. For example, the women in our participatory research activities spoke about feelings of empowerment as well as disempowerment in relation to their work in the value chain. On the one hand, women expressed some sense of empowerment through their perceived positive changes, which they connected to their involvement as processors/retailers. These positive changes included: more respect given by husbands and the community for their wage-earning status; increased household spending on foods, education, medical care, furniture, and other household investments; improved community infrastructure (water, electricity, roads) and community investments (childcare center; police station). On the other hand, the women also discussed the challenges that disempower them in their goal to be successful processors/retailers in the value chain. These barriers to their success included: a lack of support and understanding from husbands; (threat of) domestic violence or conflict (e.g., jealousy) from husbands and co-wives; lack of “freedom” to work outside of the home or a lack of mobility, tied to local religious norms and the need for a husband’s consent; a lack of transportation; and depending on one’s neighborhood, a lack of electricity, which would improve their feeling of safety when walking home from work in the dark. Despite some seemingly incompatible perceptions of empowerment and disempowerment, women’s subjectivities are an important area to explore for the VCD project to better understand the women’s needs, desires, and constraints to being processors/retailers in an important nutrition-sensitive and gender-sensitive value chain. Moreover, women’s perceptions of both empowerment and disempowerment fit with the literature’s acknowledgement that women’s empowerment is a multi-dimensional, context-specific, and complex process.

Researchers must continue to generate more evidence on gender-sensitive VCD interventions in different contexts and at different levels of analysis. Strengthening women’s participation along value chains may increase women’s empowerment along some dimensions, but there is not one solution to achieving women’s empowerment due to its complexity and, at times, contradictory processes. One woman may feel empowered by working outside of the home, but she may then feel disempowered by having less sleep if her housework duties are not shared after she becomes employed. Another woman, such as a single mother or a widow, may feel like she has no choice but to work outside of the home, even if she lacks affordable childcare while she is at work. Despite the complexity of women’s empowerment, however, the VCD projects that take a gender-equitable approach can make an important contribution to women’s lives as individuals and as collective citizens. An inclusive scaling-up of a business or value chain should involve ongoing gender analysis and research to ensure equitable access by women and men of diverse backgrounds. Understanding under what conditions VCD can positively impact women and other marginalized populations is crucial for future progress in inclusive economic development. In addition, including men and community leaders, such as religious leaders in Touba, in gender-sensitive training is valuable for VCD projects so that more people may view women’s empowerment not as disempowering to men but as empowering for women, men, children, households, and communities in the long term.

In conclusion, this study has provided insights into the perceived impacts of the VCD project on income, nutrition, and women’s empowerment. Despite the rigid gender norms in Touba, Senegal, the instant fortified flour VCD project has increased women’s participation in the value chain, as they process, package, and market an innovative and fortified product to members of their communities. Additionally, farmers associated their participation in the VCD project with increases in their financial wellbeing, and health professionals are increasingly adopting the instant fortified flours based on their perceived positive outcomes for malnourished children, women, and elderly in their communities. Based on its own assessment of the instant fortified flours, the Senegalese government’s anti-malnutrition agency (the CLM) plans to increase its purchase/subsidies of these flours from the women’s processing and retailing network. There is much opportunity to build upon and expand the VCD project’s gender research in Touba and, potentially, in a subnational study if the government’s plans to use the project as a model throughout Senegal come to fruition. Donor support for VCD projects, including the one in Touba, should include resources for more comprehensive gender-sensitive training and studies about women’s individual and collective empowerment throughout the lifetime of the projects, with an aspiration for transformative changes toward gender equality and women’s empowerment beyond the life of a project.

## Figures and Tables

**Table 1:** Processors and Retailers Descriptive Statistics.

Variable	Retailing Average	Processing Average	Overall Average	Overall Std Deviation	Obs.
Age	40.67	43.78	42.34	13.14	93
Education (years)	2.97	2.94	2.95	3.97	83
Religious member	0.95	0.78	0.86	0.35	93
Social Member	0.91	0.94	0.93	0.27	93
Union Member	0.02	0.00	0.01	0.10	93
Political Member	0.26	0.16	0.20	0.41	93
Other Member	0.00	0.00	0.00	0.00	93
Not member of any group	0.00	0.00	0.00	0.00	93

**Table 2:** Farmer Descriptive Statistics.

Variable	Obs	Mean	Std Dev	Min	Max
Education	12	3	4.2	0	10
Experience	12	33.2	15.2	10	60
Hectares farmed	11	39.1	56.52	2	200
Amount of millet harvested (tons)	12	28.1	55.5	3	200
Amount millet sold (tons)	9	125.6	180.1	3	500
Revenue USD (605 CFA/USD)	9	50,722	71,030	1240	194,215

## Data Availability

Data are archived with Purdue University’s Feed the Future Innovation Lab for Food Processing and Post-harvest Handling (FPIL). Contact FPIL’s Gender Specialist Cheryl O’Brien at cobrien@sdsu.edu.
